# Influence of Social and Psychosocial Factors on Summer Vacationers’ Sun Protection Behaviors, the PRISME Study, France

**DOI:** 10.3389/ijph.2022.1604716

**Published:** 2022-08-10

**Authors:** Cécile Durand, Anaïs Lamy, Jean-Baptiste Richard, Leïla Saboni, Florence Cousson-Gélie, Olivier Catelinois, Apolline Bord, Benoit Lepage, Damien Mouly, Cyrille Delpierre

**Affiliations:** ^1^ Regions Division Occitanie, Santé publique France (SpF), Toulouse, France; ^2^ UMR1295 CERPOP, Inserm, UPS, Université de Toulouse III, Toulouse, France; ^3^ Support, Processing and Data Analysis Division, Santé publique France (SpF), Saint-Maurice, France; ^4^ Prevention Department Epidaure, Institut du Cancer de Montpellier, Montpellier, France; ^5^ EPSYLON EA 4556, Université Paul Valéry Montpellier 3, Montpellier, France

**Keywords:** socioeconomic position, psychosocial factors, sun protection, behavior, vacationers, structural equation modelling, theory of planned behavior, ultraviolet exposure

## Abstract

**Objectives:** Summer intermittent sun exposure is a major risk factor for melanoma. Socioeconomic position, cognitive and psychosocial factors play a role in sun protection behaviors but the underlying mechanisms are unknown. This study aimed to measure the influence of educational level on sun protection behaviors in French summer vacationers on the Mediterranean coastline, and to identify the mediating psychosocial factors in this pathway.

**Methods:** In summer 2019, French vacationers aged 12–55 staying in coastline campsites were asked about their holiday sun protection behaviors, their knowledge, attitudes, perceived control, and social norm relative to sun protection. A structural equation model measured the direct and indirect effects of educational level on protection behaviors via cognitive and psychosocial factors.

**Results:** Sun protection during vacation increased with educational level. Theoretical knowledge partially mediated this association, from 22% to 86%, particularly for intermediate educational levels.

**Conclusion:** Our results highlight the importance of implementing suitable sun prevention interventions for vacationers, especially those with a lower socioeconomic position. Improving theoretical knowledge around sun protection may be an important part of broader efforts to encouraging improved preventive behaviors.

## Introduction

Melanoma is the most serious skin cancer and is primarily caused by ultraviolet (UV) radiation [1]. Incidence has steadily increased in fair-skinned populations over the last 50 years and has recently leveled off in several European countries [2]. In 2018, an estimated 290,000 melanomas with 60,000 related deaths occurred worldwide [3] with 15,500 cases and 1,975 related deaths in France [4].

The main factors involved are related to individual behaviors, making this cancer mostly preventable. More specifically, melanomas are primarily the result of inappropriate and repeated sun exposure, and a history of sunburn especially before the age of 15 [5]. Intermittent sun exposure, such as that experienced during recreational exposure and summer holidays, means vacationers seeking sunny and warm destinations are particularly vulnerable [5]. In fair-skinned European populations, overexposure is partially driven by vacationers’ desire to tan, since a moderate tan is still a positive social norm associated with beauty, health and well-being [6, 7].

Modifiable and non-modifiable factors play a role in protection behaviors [8]. In terms of non-modifiable factors, young age, male sex, race or ethnicity (white, non-hispanic), skin sensitivity, place of residence (sunny area), and personal or family history of melanoma are all determinants of sun protection. Socioeconomic position (SEP) (educational level, occupation, income, etc.) is mostly positively associated with protection behaviors [8–10]. Bocquier et al. [11] found this association to be both direct and indirect through the mediation path of knowledge. In terms of modifiable factors, knowledge, attitude, risk-awareness [12], self-efficacy [13] and, finally, intentions [14] were associated with protection behaviors in previous studies [8]. The Theory of Planned Behavior (TPB) [15] has already been used to model sun protection behaviors. Its main determinants - attitude, perceived control and social norm–have been associated with intention to protect oneself and with behaviors. Specifically, the TPB explained 39% and 25% of variance in intentions and behaviors, respectively in one meta-analysis [16].

Despite annual public health campaigns since 1996, French people continue to overexpose themselves without comprehensive skin protection. Some persistent misconceptions about sun exposure and protection are growing, for example concerning consequences of sunburns in childhood or concerning photoaging [17]. With a high level of UV radiation and millions of vacationers each summer, the French Mediterranean coastline is a particularly relevant place to study sun exposure behaviors of vacationers and their determinants. Factors found to be associated to sun protection in the general population by the international literature need to be confirmed in the French summer vacationer population, and knowledge about mechanisms by which French summer vacationers engage in protective behaviors need to be improve with a view to developing more effective prevention messages, and identifying new targets for prevention interventions.

For this purpose, the PRISME (PRevention and Impact of Sun exposure on the French MEditerranean coast) study was implemented in 2019 [18] and the role of cognitive and psychosocial mediation variables in the causal pathways from SEP to protection was analyzed in order to build appropriate interventions that do not increase social health inequalities.

So, the objectives of the present analysis were 1) to measure the influence of educational level on sun protection behaviors in French summer vacationers on the Mediterranean coastline, and 2) to identify the mediating psychosocial factors in this pathway, using structural equation modelling.

## Methods

### Material

#### Study Design

This study is part of a larger cluster randomized crossover trial named PRISME. A detailed description of the PRISME methodology (sample size, intervention, randomization method) was previously published [18]. Briefly, baseline (T0) and first follow-up (T1, 4 days later) took place in eight campsites from 7 July to 30 August 2019 along the Occitanie Mediterranean coastline (south of France). The second follow-up (T2) took place online between October and November 2020. Two previously described sun prevention interventions [18] were delivered to some of the participants at T0 (just after the baseline data collection). In this study, only data from the T0 and T1 were used and were spaced 4 days apart. At T0, in each campsite each week, a two-stage sampling permitted to randomly drawn first the pitches, and second the individuals.

#### Participants: Inclusion Criteria

The target population was French vacationers 12–55 years old staying in campsites along the Mediterranean coastline. Inclusion criteria were as follows: French speaking, living in France, no health problems which completely precluded sun exposure, staying at least 4 days in one of the eight selected campsites, and for minors, staying in the campsite with a legal guardian.

#### Data Collection and Questionnaire

T0 and T1 standardized questionnaires were administered face-to-face [18]. The T0 questionnaire collected sociodemographic and physical data, items to measure cognitive factors (knowledge and misconceptions about sun exposure and protection), and the following psychosocial factors which contribute to the process of change as per the TPB [15]: attitudes toward sun exposure and tanning, influence of relatives (social norm) and perceived behavioral control. The T1 questionnaire included items measuring protection behaviors during the 4 days since T0. Items focusing on knowledge, attitudes, and behaviors were adapted from the annual French Health Barometer survey questionnaire [17] and international literature [19–22].

### Construction of Variables

#### Outcome

Among the various possible sun protection measures [23, 24], we measured behaviors both by the use of sun protection resources and by the limitation of exposure, in accordance with current prevention recommendations. This variable was constructed from six items, adapted from Glanz [22], measured at T1 using the same 5-point Likert scale (never = 0/rarely = 1/sometimes = 2/often = 3/always = 4). These items measured the declared frequency of the following recommended behaviors when staying outdoors for more than 15 min during their vacation and since T0: 1) wearing a t-shirt that covered their shoulders, 2) a hat, 3) sunglasses, 4) putting on sunscreen every 2 hours, 5) staying in the shade, and 6) avoiding high-risk hours (i.e., noon to 4 p.m.).

#### Predictor

SEP is multidimensional and three indicators are frequently used to measure it: education, occupation and income [25, 26]. Educational level seemed to us to be the best indicator for our analysis because it was a hierarchical and stable life course indicator associated with many health behaviors. Moreover, it is a distal measure of early life SEP and an antecedent to the proximal measures of occupation and income [25–27]. In our data, educational level was coded 0 = less than secondary school certificate, 1 = secondary school certificate, 2 = 1- to 2-year university diploma, 3 = 3-year (bachelor) or 4 = 4-year university degree or higher. For minors, the highest certificate obtained by either one of their parents was used.

#### Potential Mediators

Three items collected attitudes toward sun-exposure and sun tanning using the same 5-point Likert scale (0 = strongly agree, 1 = tend to agree, 2 = neither agree nor disagree, 3 = tend to disagree, 4 = strongly disagree): “*I like to sunbathe*”, “*I think I am more beautiful when I am tanned*”, “*I feel better when I am in the sun*”. Higher points meant less favorable attitudes.

Social norm was measured with two items on a reverse 5-point Likert scale (0 = strongly disagree to 4 = strongly agree): *The people who I care about:* “*encourage me to protect myself from the sun*”* / *“*protect themselves from the sun*”.

Perceived behavioral control was measured with one item: “*During vacation, protecting myself from the sun is very difficult* (=0) *difficult* (=1), *neither difficult nor easy* (=2), *easy* (=3), *very easy* (=4).

Knowledge about sun prevention was composed of two dimensions: 1) theoretical knowledge of sun protection recommendations and harmful consequences of sun exposure (Knowledge 1), and 2) misconceptions about sun protection and exposure (Knowledge 2):

- Theoretical knowledge (Knowledge 1) was measured with four items:
  a) The number of recommended sun protection behaviors cited (staying in the shade, wearing a t-shirt, a hat, sunglasses, sunscreen, avoiding high-risk hours),
  b) The number of harmful consequences of intense exposure cited among the main negative effects (sunburn, sunstroke/heatstroke, sun-related rashes, eye problems, skin cancer, photoaging),
  c) Knowledge of high-risk hours (0 = none cited between noon and 4 pm / 1 = some cited between noon and 4 pm / 2 = all cited between noon and 4 p.m. but also other hours cited / 3 = all cited between noon and 4 p.m. exclusively),
  d) Knowledge of the recommended frequency for applying sunscreen (0 = less than once every 2 h / 1 = more than once every 2 h / 2 = every 2 h (official recommendation)).
- Misconceptions (Knowledge 2) were measured with five items on a 5-point Likert scale (0 = Strongly agree to 4 = strongly disagree): “*I can sunbath longer with sunscreen*”, “*Sunburn prepares the skin for the sun*”, “*If the weather is cloudy, I have to protect myself from the sun*”, “*Sunburns in childhood have consequences in adulthood*”, “*Exposure to the sun will make my skin wrinkle sooner than expected*” (reverse scale for the last 3 items). Higher points meant fewer misconceptions.

“I don’t know” answers were scored as “neither agree nor disagree” for social norm (<0.5%) attitudes (<0.5%) and for Knowledge 2 items (<0.5%–5.2%).

#### Adjustment Variables

Confounders corresponded to individual factors associated with sun protection according to the literature, and potentially associated with education.

Skin sensitivity was evaluated using six characteristics: skin, eye and hair color, presence of moles, tendency to sunburn, and tendency to tan after a critical exposure. To avoid subjectivity in phototype classification [28–30], we created homogeneous classes using a multiple correspondence analysis (MCA) followed by a hierarchical ascendant classification (HAC). This led to a 4-group classification, consistent with the Fitzpatrick phototype and with colorimetry data collected from the participants [18] (Supplementary File S1), which coded highly sensitive skin, sensitive, slightly sensitive (reference group) and dark to black skin.

Participant age was coded into five classes: 12–14, 15–24 (ref.), 25–34, 35–44 and 45–55 years old. Sex was coded into man (ref.) and woman.

Personal or loved one’s history of cancer meant the participant or a loved one, friend or colleague, had been or was currently being treated or followed for skin cancer.

The analysis were also adjusted on study design variables potentially associated with protection behaviors: campsite because of difference in populations, week of inclusion because of weather, time since arrival because of possible change in behaviors along holidays, and intervention group since the interventions were delivered before the measure of the outcome. The reference campsite was the one with the largest sample (campsite 4). The reference week was in the middle of summer (calendar week 31). Time since campsite arrival was a continuous variable (in days).

### Statistical Analysis

The means of the six items (0–4) included in the protection outcome were calculated stratified by individual factors, and simple regressions models were performed to measure the univariate association.

**FIGURE 1:**
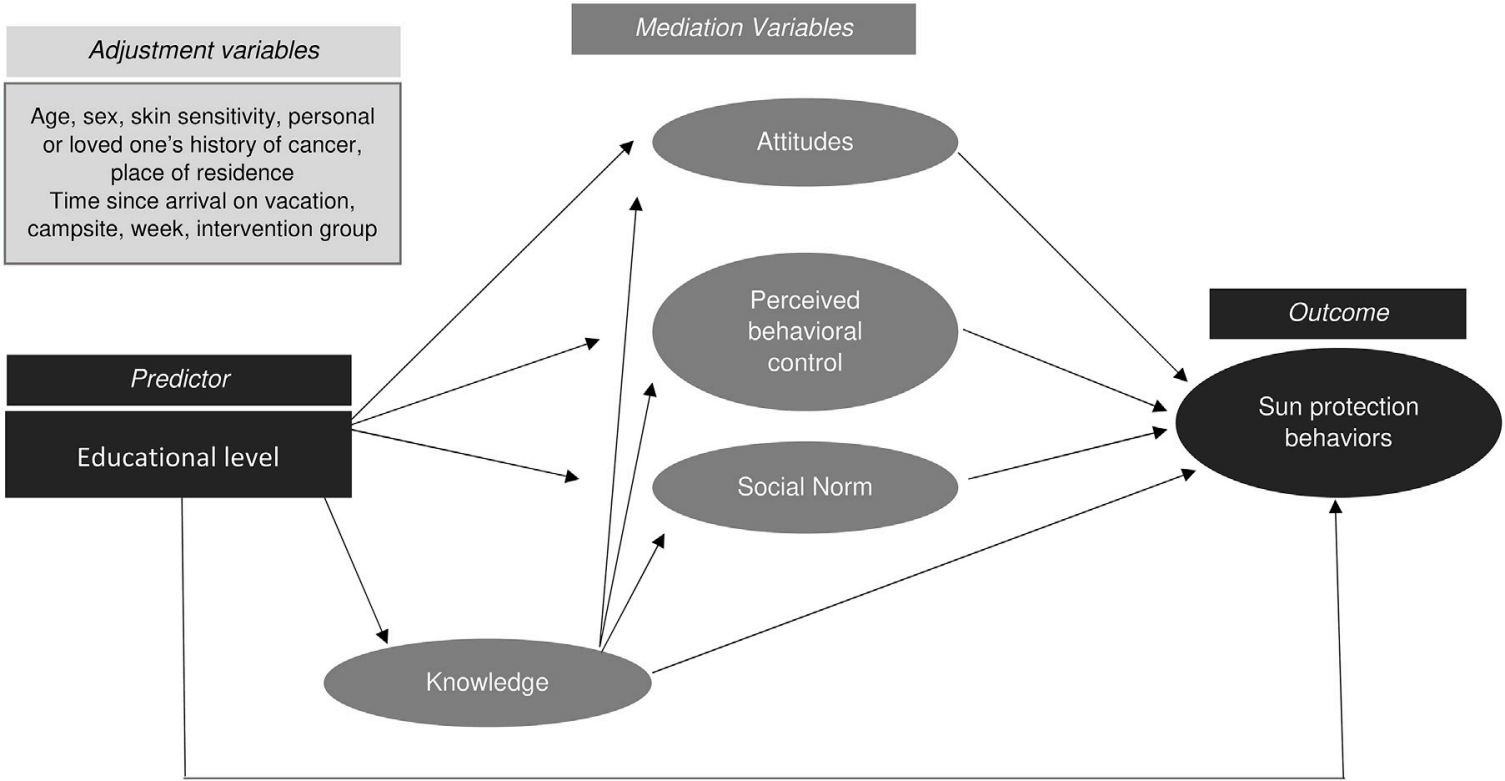
Conceptual model to analyze the mediation paths between educational level and sun protection behaviors - PRISME, France, 2019.

To study the possible mediation paths between educational level and sun protection behaviors, a conceptual model was constructed (Figure 1) in accordance with the TPB, whereby behaviors are influenced by psychosocial determinants, which in turn are influenced by knowledge and other individual factors, including educational level and confounders [15]. We hypothesized that knowledge is an intermediate mediator between educational level and the main determinants of the TPB, given the probable link between education and knowledge.

Because Knowledge 1 and 2, attitudes, social norm, and the outcome were all measured by several items, we constructed latent variables. To do this, we used Cronbach’s alpha coefficient [31], MCA graphs analysis and a confirmatory factor analysis (CFA) [32]. These analyses concluded that while some items contributed less to the latent variable construct, each item contributed significantly and was able to measure the overall concept. (Supplementary File S2).

We then fitted a model using structural equation modelling (SEM) to estimate beta coefficient parameters and their standard errors using the Taylor linearization method because of the complex sample design [33]. SEM takes into account multiple and interrelated dependence relationships in a system of simultaneous equations, and can represent unobserved concepts with latent variables adjusting for measurement errors in the estimation process [34–36].

All direct associations represented by arrows in Figure 1 were estimated after adjustment for confounders and study design variables. Non-significant associations were removed from the model. For educational level, a significant association with only one of the educational level categories was sufficient to maintain the link in the model. For each mediation path (i.e., a succession of significant associations from educational level to the outcome), the indirect effect of educational level on the outcome was estimated. The sum of all indirect effects and the direct effect equaled the total effect of educational level on protection behaviors.

Goodness-of-fit of the final model was assessed by the root mean square error of approximation (RMSEA), the standardized root mean square residual (SRMR), and the coefficient of determination (CD). RMSEA<0.06 and SRMR<0.08 were considered a reasonable fit [34].

All descriptive and analytical analyses were performed on weighted data by the probability of inclusion at each of the two-stage sampling levels. Statistical significance was defined by a two-sided *p-*value < 0.05 (noted *p < 0.05, **p < 0.01, ***p < 0.001). The analyses were performed using Stata version 14.2 and R-studio version 1.3.

## Results

The study included 1,355 participants at T0 and 1,283 were followed up at T1. Average age was 32.6 years old, 52% were women, 65% did not have a university diploma, and 80% had sensitive or highly sensitive skin (Table 1). A few missing data were observed and complete data were available for 1,267 participants (98.8%).

**TABLE 1:** Sun protection behaviors items according to demographic, socioeconomic and physical factors—univariate analysis (*n* = 1,283)–PRISME, France, 2019.

			Means of protection Behaviors items (coded 0–4)^a^					
	n	% (weighted)	Stay in the shade	Avoid noon-4Pm	Use of sunscreen	Use of sunglasses	Use of hat	Use of t-shirt
**Age**			***p<0.001***	***p<0.001***	*NS*	***p<0.001***	***p<0.001***	***p<0.001***
12–14 years	191	11.6	2.1	2.3	2.4	1.6	1.8	2.1
15–24 years	277	24.7	1.6	2.0	2.1	2.4	1.4	1.4
25–34 years	156	13.6	2.2	2.5	2.6	2.9	1.6	1.7
35–44 years	299	25.9	2.5	2.8	2.5	3.2	2.1	1.8
45–55 years	360	24.3	2.6	2.9	2.4	3.2	2.2	2.0
**Sex**			*NS*	*NS*	***p<0.001***	***p = 0.002***	***p = 0.010***	***p<0.001***
Men	560	47.7	2.2	2.5	2.0	2.6	2.0	2.1
Women	723	52.3	2.3	2.5	2.7	3.0	1.7	1.4
**Educational level**			*NS*	***p = 0.003***	***p<0.001***	***p = 0.018***	***p = 0.022***	*NS*
Less than secondary school certificate	406	32.9	2.2	2.3	2.1	2.6	1.8	1.7
Secondary school certificate	384	32.1	2.2	2.5	2.3	2.7	1.7	1.7
1 or 2-year university diploma	213	16.5	2.3	2.7	2.6	3.1	1.8	1.6
3-year university diploma	134	10.1	2.2	2.5	2.7	3.0	2.2	1.9
4-year university diploma or higher	138	8.5	2.2	3.0	3.0	3.2	2.4	2.1
**Skin sensitivity**			***p<0.001***	*p = 0.073*	***p<0.001***	*NS*	***p<0.001***	***p<0.001***
Highly sensitive	438	33.7	2.4	2.7	2.6	2.9	2.1	2.0
Sensitive	583	46.2	2.2	2.5	2.4	2.8	1.9	1.7
Slightly sensitive	213	15.6	1.7	2.4	1.9	2.7	1.2	1.3
Dark to black skin	49	4.6	2.3	1.9	2.6	2.8	1.9	2.2
**Personnal or loved one’s history of cancer**			*NS*	***p = 0.031***	*NS*	*NS*	*NS*	*p = 0.077*
Yes	198	15.4	2.2	2.8	2.5	2.9	1.9	2.0
No	1,085	84.6	2.2	2.5	2.4	2.8	1.9	1.7

a 0 = Never / 1 = Rarely / 2 = Sometimes / 3 = Often / 4 = Always.

NS = not significant.Bold-italic represents *p*-values < 5%.

The mean of each protection item included in the outcome latent variable highlighted that people with a lower educational level reported using the protection resources less frequently (except wearing a t-shirt) and avoiding sun exposure less during high-risk hours. Moreover, 15–24 year-olds were the group which exposed themselves most and used protective resources least frequently, except for sunglasses as 12–14 year-olds declared even less frequent use. The protection resources used differed between women and men; women reported using sunglasses and sunscreen more frequently but hats and t-shirts less frequently than men. People with slightly sensitive skin declared using protection resources (except sunglasses) less frequently and spending more time in the sun than others (Table 1).

**FIGURE 2:**
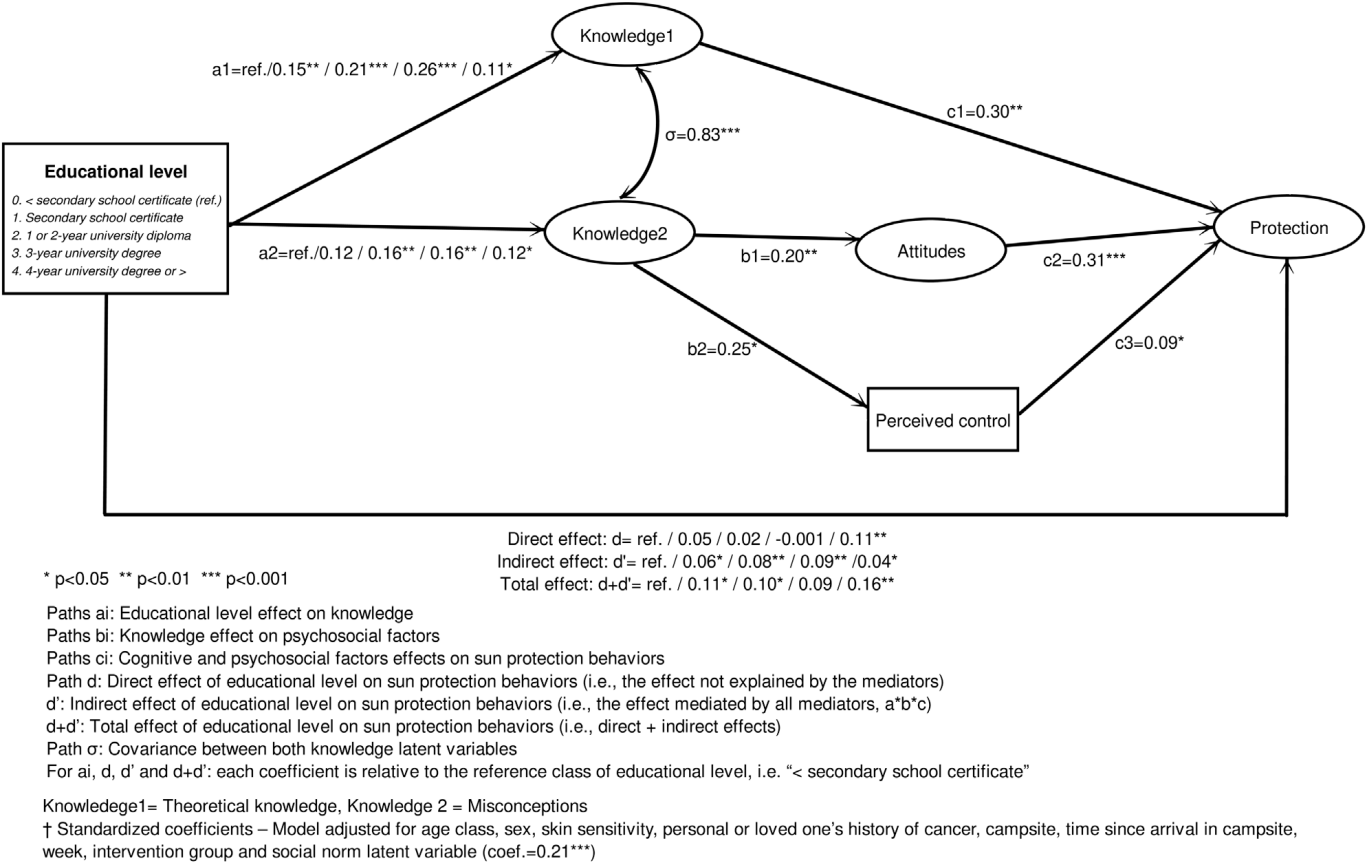
Effects of educational level on sun protection behaviors via cognitive and psychosocial factors, estimated using a structural equation model^†^ (*n* = 1,267)–PRISME, France, 2019.

The direct global effect of educational level was not significant (*p* = 0.084) but vacationers with a 4-year university degree or higher had a greater level of protection than the ‘less than secondary school certificate’ group (*ß* = 0.11**). Moreover, educational level was associated with Knowledge 1 (*ß* = ref./0.15**/0.21***/0.26***/0.11*, *p* < 0.001) and Knowledge 2 (*ß* = ref./0.12/0.16**/0.16**/0.12*, *p* = 0.016) (Figure 2).

Considering cognitive and psychosocial factors, theoretical knowledge (Knowledge 1: *ß* = 0.30, *p* = 0.002), attitudes (*ß* = 0.31, *p* < 0.001), perceived behavioral control (*ß* = 0.09, *p* = 0.036), and social norm (*ß* = 0.21, *p* = 0.003) were all directly associated with protection behaviors. Misconceptions (Knowledge 2) were not directly associated with the outcome but were significantly associated with attitudes (*ß* = 0.20, *p* = 0.006) and perceived control (*ß* = 0.25, *p* = 0.012) (Figure 2).

Accordingly, we were able to explore three mediation paths (indirect effect) from educational level to protection:

1) *via* Knowledge 1: *ß* = ref./0.05/0.06*/0.08**/0.03 (*p* = 0.073).
2) *via* Knowledge 2 and attitude: *ß* = ref./0.01/0.01*/0.01*/0.01 (*p* = 0.353).
3) *via* Knowledge 2 and perceived control: none of the indirect effect coefficients were significant (*p* = 0.621).

Finally, the direct effect of educational level on protection was modified by adding the indirect effects (*ß* = ref./0.06*/0.08**/0.09**/0.04*, *p* = 0.023). The total effect was clearly significant with protection increasing with educational level at two cut-off levels: secondary school certificate and 4-year university degree or higher (*ß* = ref./0.11*/0.10*/0.09/0.16**, *p* = 0.009).

With regard to this association between education and protection, 28%–100% (52%/77%/100%/28%) of the total effect was mediated, particularly via theoretical knowledge (43%/64%/86%/22%). This mediated path was less strong in persons with a 4-year university degree or higher (Table 2).

**TABLE 2:** Direct and indirect effects of educational level on sun protection behaviors estimated using a structural equation model (n = 1,267)–PRISME, France, 2019.

Comparing with Less than secondary school certificate		Secondary school certificate			1 or 2-year university diploma			3-year university degree			≥4-year university degree		
Effect	Via	Coef.^a^	CI95%	%	Coef.^a^	CI95%	%	Coef.^a^	CI95%	%	Coef.^a^	CI95%	%
indirect	→ Theoretical knowledge	0.05	[−0.00–0.09]	43	0.06*	[0.01–0.12]	64	0.08**	[0.02–0.14]	86	0.03	[−0.00–0.07]	22
indirect	→ Misconceptions → Attitudes	0.01	[−0.00–0.02]	7	0.01*	[0.00–0.02]	10	0.01*	[0.00–0.02]	11	0.01	[−0.00–0.02]	5
indirect	→ Misconceptions → Perceived control	0.00	[−0.00–0.01]	2	0.00	[−0.00–0.01]	3	0.00	[−0.00–0.01]	4	0.00	[−0.00–0.01]	2
Indirect total		0.06*	[0.00–0.11]	52	0.08**	[0.02–0.13]	77	0.09**	[0.03–0.15]	100	0.04*	[0.00–0.09]	28
Direct		0.05	[−0.05–0.15]	48	0.02	[−0.08–0.12]	23	0.00	[−0.10–0.10]	0	0.11**	[0.03–0.20]	72
**Total direct + indirect**		0.11*	**[0.01–0.21]**	**100**	0.10*	**[0.00–0.20]**	**100**	**0.09**	**[-0.00–0.19]**	**100**	0.16**	**[0.06–0.26]**	**100**

a Standardized coefficients.

*p < 0.05 **p < 0.01 ***p < 0.001.

With respect to confounders, the direct effect of age in this fully adjusted model was significant (*ß* = 0.19***/ref./0.17**/0.32***/0.38***, *p* < 0.001) with the lowest protection observed in 15–24 year-olds, followed by the 12–15 and 25–34 year-olds. An increase in protection was observed between persons 25 to 55 years old. Increased skin sensitivity was associated with an increase in protection (*ß* = 0.25**/0.21**/ref./0.12, *p* = 0.011). Protection was also associated with the campsite (*p* = 0. 009). None of the other adjustment variables was statistically associated with protection (sex *p* = 0.850, history of cancer *p* = 0.843, place of residence *p* = 0.148, time since arrival *p* = 0.319, week *p* = 0.176, intervention group *p* = 0.556).

Goodness-of-fit for the final SEM model was good: RMSEA = 0.037, SRMR = 0.037, CD = 0.94.

## Discussion

Our study showed that the educational level was indirectly associated with sun protection behaviors via theoretical knowledge, and to a lesser extent via misconceptions and attitudes toward exposure and tanning, especially for intermediate educational levels (1 to 3-year university diploma). After taking into account the mediation paths, a direct association persisted for the highest educational level (4-year university degree or higher) and remained unexplained by the analyzed mediators.

The indirect effect accounted for 28% to 100% of the total effect, depending on educational level categories. Theoretical knowledge contributed to this association, since it mediated 22% to 86% of the total effect, particularly in intermediate educational levels. On the contrary, the large direct effect measured among participants with the highest educational level suggests either a lack of statistical power in this category, or that other mediating factors were not measured in this study and so unidentified mechanisms could been at work in this subpopulation and need to be evaluated in future studies.

Our results also found direct associations between protection and certain confounders, particularly age and skin sensitivity. Consistent with the TPB [15], protection behaviors were also associated with latent variables measuring attitudes, perceived behavioral control, and social norm, as well as theoretical knowledge. Misconceptions were also indirectly associated through attitudes and perceived control.

Our study confirms some results found in the literature and brings new elements of understanding of sun protection behaviors. Our results adds to evidence from previous studies [8, 9, 11] which showed that less socially advantaged persons are less likely to engage in sun protection behaviors, just as is the case for other health behaviors associated with a greater risk of cancer. As new findings, we identified the mediation paths that contribute to this association, through knowledge, but also through misconceptions and attitudes toward tanning, and highlighted the importance of the general public’s knowledge in the context of increasing sun protection behaviors, especially in persons with intermediate educational levels.

More generally, negative attitudes toward tanning were strongly associated with protection behaviors, which is consistent with previous studies [37]. This result supports our initial hypothesis [18] of the importance to lessen the vacationers’ attraction for tanning, and to deliver appearance-based arguments in preventive interventions.

Our study also confirms the association of the TPB factors attitude, perceived control, and social norm, with sun protection behaviors [16]. However, in the TPB, these psychosocial factors are linked to behaviors via intentions. In our study, intentions to protect oneself from the sun during vacation were also measured at T0 but were not included in our model. Intentions at T0 and behaviors at T1 were strongly correlated (correlation = 0.71 between the two sum-scores) because the time between both measures was very short (4 days), leading to non-convergence of the model when both latent variables were introduced simultaneously. Additional SEM models fitted with the “intentions” latent variable alone, and with “intentions” and “behaviors” as sum-scores rather than latent variables, provided similar findings. Therefore, TPB psychosocial factors contributed to sun protection behaviors, and we can hypothesize that this association is largely mediated by intentions.

One possible limitation of our study is that the summer vacationer population was selected from only one type of tourist accommodation, specifically campsites. However, campsites represent a large part of summer tourist accommodation on the Mediterranean coast (63% according to [38]) and host populations varied in age, sex and socioeconomic position. Although, few data exist to compare our population to the overall French summer vacationer population, it is reasonable to assume that we can generalize our results to the more than three million French summer vacationers who stay in campsites along the Mediterranean coast each summer.

A probable second limitation is reporting bias. The data used came from declarative questionnaires. Such self-reported data, notably items dealing with behaviors, are particularly prone to social desirability bias [24, 39] and additional research is needed in the field of sun protection behaviors to determine if this bias is a differential bias according to individual characteristics such as SEP.

Moreover, most of our items were constructed from similar studies. However, the constructed variables used are not validated scales, and this may have led to measurement bias. Some items had low alpha and factor loading, reflecting an imperfect construct. However, the different methods used to evaluate the correlation of items at different collection times provided consistent results. Furthermore, the use of latent variables in the SEM analysis helped limit these measurement errors somewhat [34–36].

Furthermore, the predictor may have been imperfect. Educational level may be too restrictive a dimension to represent the complexity of a person’s entire socioeconomic position [25, 26]. Measuring persons’ social and economic positions, as well as cultural heritage, requires more complex indicators than those we examined.

Finally, caution is required in any causality analysis using SEM models. The associations represented by unidirectional arrows make strong assumptions about the chronology of events and interpretation must be based on theoretical models. The links found may indeed represent a reverse causality, especially when using cross-sectional data. In our data, while this question did not really arise for confounders which were intrinsic individual characteristics present long before the stay, it was crucial when examining causality between the mediating variables and the protection outcome. For example, it is not clear whether a high level of knowledge led to a high level of protection or the opposite. To limit this risk, we based our conceptual model on the TPB, which is an already proven model for the analysis of sun protection behaviors [16]. Moreover, we used longitudinal data with an outcome measured 4 days after the other variables in order to better control for the chronology of the events. However, because the time between measures was very short and measures at T0 and T1 were strongly correlated, it is likely our data faced the same limitations as cross-sectional data and reverse causality was still possible.

Our results have several consequences for sun prevention in terms of which populations to target and intervention mechanisms to activate. For the former, sun-preventive interventions for vacationers should be particularly oriented towards the youngest populations, people with sensitive skin, those with lower educational levels, and more broadly, towards less socially advantaged people. As these factors are not modifiable, the mechanisms to be implemented to reach these populations need greater investigation. However, it seems that, the cognitive and psychosocial factors present in TPB are a possible lever for improving sun protection. Interventions that increase knowledge, minimize attitudes which favor of tanning and sun exposure, build self-efficacy, and create a social environment that encourages protection, could be effective. With respect to populations with a low level of education, implementing interventions that do not increase social inequalities is the real challenge. Although additional mechanisms are needed to turn theoretical knowledge into real behavioral change in preventive interventions [40, 41], improving knowledge about sun exposure and protection may be an important part of broader efforts to encouraging improved sun preventive behaviors. In order to reach the less socially advantaged vacationers, future sun preventive interventions will need to pay attention to literacy by adapting the messages and information media, and to target working-class location like beachfronts or campsite rather than airports or luxury resorts.
