# Supplementary material for: Influence of Social and Psychosocial Factors on Summer Vacationers’ Sun Protection Behaviors, the PRISME Study, France
Source: Int J Public Health. 2022 Aug 10;67:1604716. doi: 10.3389/ijph.2022.1604716 (PMC9399345; doi:10.3389/ijph.2022.1604716)
Supplement: Supplementary file 1 [file DataSheet2.pdf]

International Journal of Public Health

Influence of social and psychosocial factors on French summer vacationers' sun protection behaviors, the PRISME study, France

Supplementary file 2: Construction of latent variables

To construct latent variables, for each group of items corresponding to a given latent variable, correlations between items were measured using Cronbach's alpha coefficient (an alpha>0.70 may be considered acceptable but the number of items needs to be considered) (1). MCA were performed and related graphs visually analyzed to confirm the proximity of answers between items. A confirmatory factor analysis (CFA) with all latent variables was then performed to measure the factor loadings of each item (i.e., their contribution to the variance of the related latent variable). In general, a factor loading ≥0.50 may be considered acceptable to conclude to a convergent validity of the construct (2).
As these items were measured at T0, T1 and T2, we calculated the alpha and the factor loadings for each latent variable at the two other data collection times.

Sun protection behaviors during the stay (at T1)

This variable was constructed from six items, measured at T1 using the same 5-point Likert scale (never=0 /rarely=1 /sometimes=2 /often=3 /always=4) by the following question:
On sunny days since our last meeting, have you used the following methods to protect yourself from the sun when you've been outside for more than 15 minutes
a) staying in the shade or under a parasol
b) avoiding sunny hours between 12 p.m. and 4p.m.
c) putting on sunscreen every 2 hours
d) wearing sunglasses
e) wearing a hat or cap
f) wearing a t-shirt that covers the shoulders

Table S2.1. Internal consistency of the constructed latent variable 'Sun protection behaviors' in terms of Cronbach's alpha, correlation, and factor loading of the CFA[a] – PRISME, France, 2019

	N	Alpha[b]	Correlation with the rest	CFA[a] Factor loading
**Sun protection behaviors (at T1)**	**1279**	**0.63**		
Stay in the shade	1282	0.55	0.44	0.59***
Avoid 12-4pm	1282	0.57	0.40	0.53***
Use sunscreen	1280	0.61	0.28	0.31***
Wear sunglasses	1282	0.60	0.31	0.34***
Wear a hat	1281	0.55	0.44	0.50***
Wear a t-shirt	1282	0.61	0.27	0.50***

[a] CFA : Confirmatory factor analysis

[b] Cronbach's alpha for the total group and with deletion of each of the individual items

*p<0.05, **p <0.01, ***p <0.001

Additionally, at T0 and T2, alpha were respectively 0.63 and 0.69. Factor loadings were all significant (p<0.001) and respectively between 0.31-0.66, and 0.35-0.77.

Graph S2.1. Multiple correspondence analysis graph representing the projection of the modalities of the items included in sun protection behaviors latent variable - PRISME, France, 2019

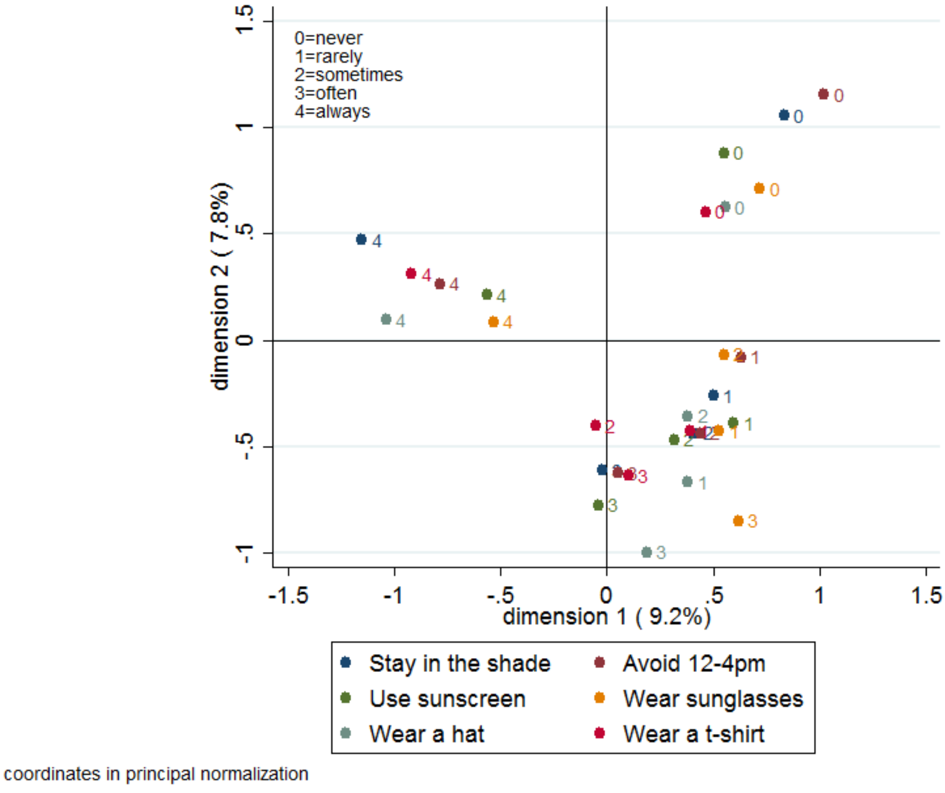

Theoretical knowledge - Knowledge 1 (at T0)

Theoretical knowledge (Knowledge 1) was measured with four items:
a) The number of recommended sun protection behaviors spontaneously cited (staying in the shade, wearing a t-shirt, a hat, sunglasses, sunscreen, avoiding high-risk hours) (*Nb protection*),
b) The number of harmful consequences of intense exposure spontaneously cited among the main negative effects (sunburn, sunstroke/heatstroke, sun-related rashes, eye problems, skin cancer, photoaging) (*Nb consequences*).
c) Knowledge of high-risk hours (0=none cited between noon and 4 pm /1=some cited between noon and 4 pm /2=all cited between noon and 4 pm but also other hours cited /3=all cited between noon and 4 pm exclusively) (*High-risk hours*),
d) Knowledge of the recommended frequency for applying sunscreen (0= less than once every 2 hours /1=more than once every 2 hours /2=every 2 hours (official recommendation)) (*Sunscreen frequency*),

Table S2.2. Internal consistency of the constructed latent variable 'Knowledge 1' in terms of Cronbach's alpha, correlation, and factor loading of the CFA[a] – PRISME, France, 2019

	N	Alpha[b]	Correlation with the rest	CFA[a] Factor loading
**Knowledge 1 (at T0)**	**1355**	**0.51**		
Nb. Protection	1355	0.37	0.37	0.52***
Nb. consequences	1355	0.40	0.34	0.53***
High-risk hours	1355	0.45	0.29	0.48***
Sunscreen frequency	1355	0.52	0.21	0.29***

[a] CFA : Confirmatory factor analysis

[b] Cronbach's alpha for the total group and with deletion of each of the individual items

*p<0.05, **p <0.01, ***p <0.001

Additionally, at T1 and T2, alpha were respectively 0.55 and 0.63. Factor loadings were all significant (p<0.001) and respectively between 0.26-0.64, and 0.33-0.81.

Graph S2.2. Multiple correspondence analysis graph representing the projection of the modalities of the items included in Knowledge 1 latent variable - PRISME. France. 2019

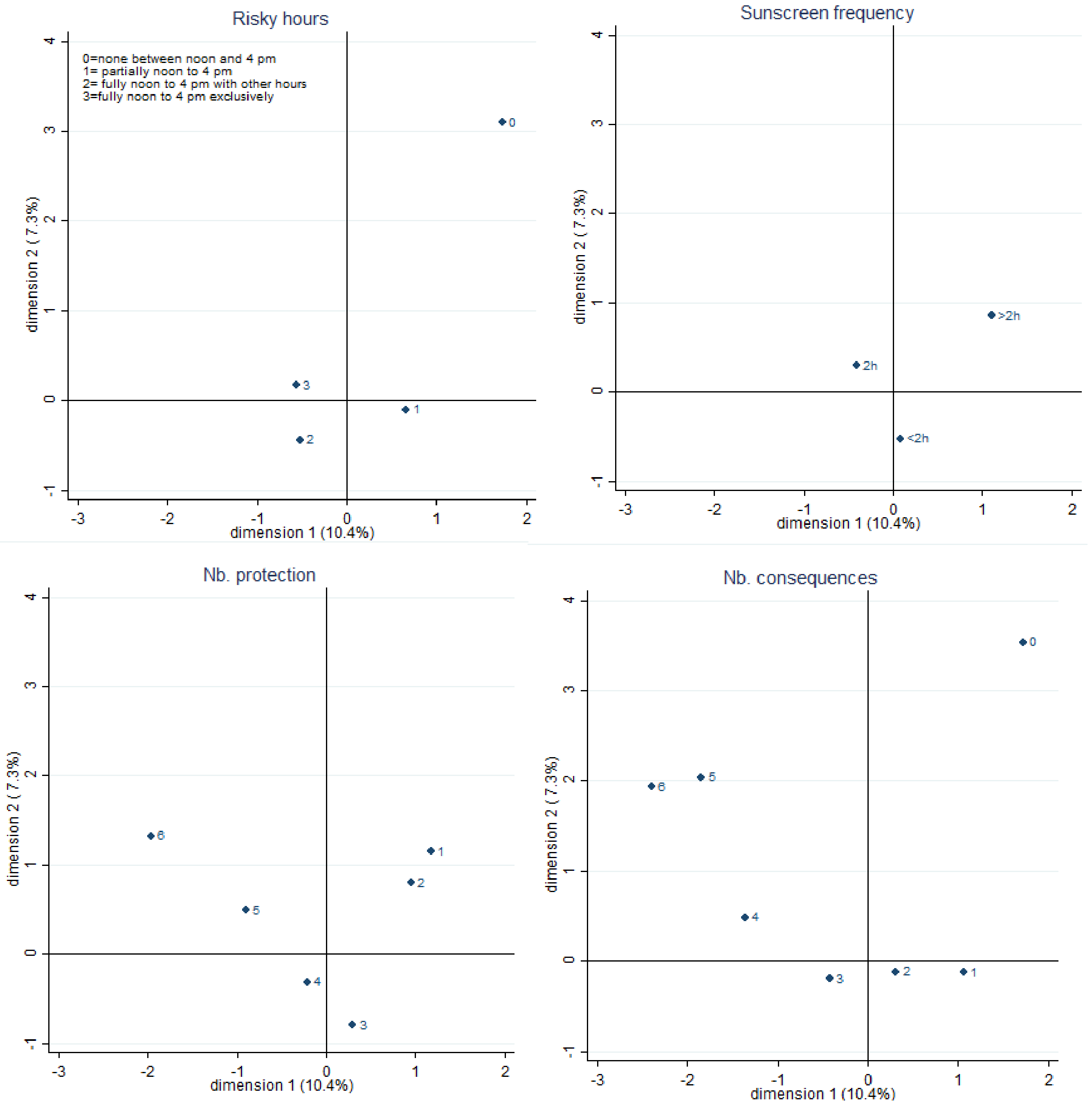

Misconceptions - Knowledge 2 (at T0)

Misconceptions (Knowledge 2) were measured with five items on a 5-point Likert scale (0=Strongly agree to 4=strongly disagree):

- “*I can sunbath longer with sunscreen” (Sunscreen exposure time +)*,
- *“Sunburn prepares the skin for the sun” (Sunburn prepare the skin),*
- *“If the weather is cloudy, I have to protect myself from the sun” (Protection when cloudy),*
- *“Sunburns in childhood have consequences in adulthood” (Sunburn consequences adult),*
- *“Exposure to the sun will make my skin wrinkle sooner than expected” (Photoaging)* (reverse scale).

Table S2.3. Internal consistency of the constructed latent variable ‘Knowledge 2’ in terms of Cronbach’s alpha, correlation, and factor loading of the CFA[a] – PRISME, France, 2019

	N	Alpha[b]	Correlation with the rest	CFA[a] Factor loading
**Knowledge 2 (at T0)**	**1354**	**0.58**		
Sunscreen exposure time +	1354	0.59	0.22	0.25***
Protection when Cloudy	1355	0.49	0.41	0.59***
Sunburn prepare the skin	1355	0.54	0.30	0.37***
Sunburn consequences adult	1355	0.51	0.37	0.55***
Photoaging	1355	0.49	0.40	0.55***

[a] CFA : Confirmatory factor analysis

[b] Cronbach's alpha for the total group and with deletion of each of the individual items

*p<0.05, **p <0.01, ***p <0.001

Additionally, at T1 and T2, alpha were respectively 0.56 and 0.60. Factor loadings were all significant (p<0.01) and respectively between 0.20-0.65, and 0.28-0.75.

Graph S2.3. Multiple correspondence analysis graph representing the projection of the modalities of the items included in Knowledge 2 latent variable - PRISME, France, 2019

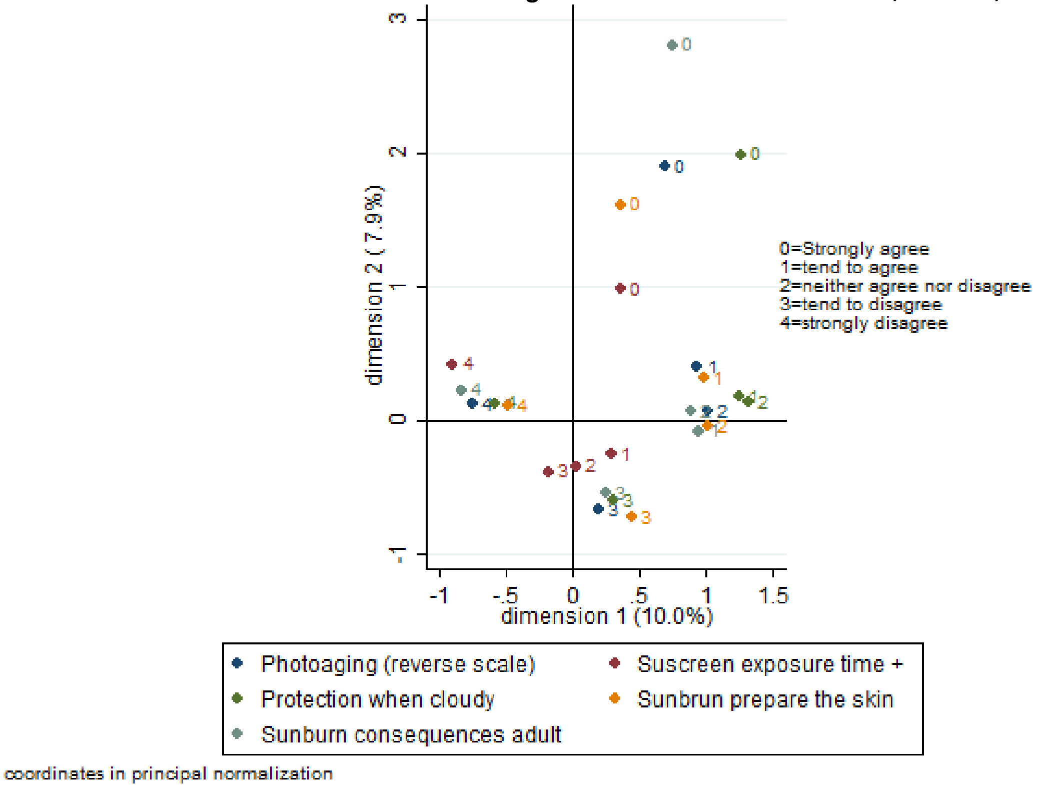

Attitudes (at T0)

Three items collected attitudes toward sun-exposure and sun tanning using the same 5-point Likert scale (0=strongly agree, 1=tend to agree, 2=neither agree nor disagree, 3=tend to disagree, 4=strongly disagree): *"I like to sunbathe"* (Like to sunbathe), *"I think I am more beautiful when I am tanned"* (Tan attractive), *"I feel better when I am in the sun"* (Exposure well-being).

Table S2.4. Internal consistency of the constructed latent variable 'Attitudes' in terms of Cronbach's alpha, correlation, and factor loading of the CFA[a] – PRISME, France, 2019

	N	Alpha[b]	Correlation with the rest	CFA[a] Factor loading
**Attitudes (at T0)**	**1355**	**0.65**		
Like to sunbathe	1355	0.46	0.52	0.94***
Tan attractive	1355	0.59	0.43	0.49***
Exposure well-being	1355	0.60	0.42	0.51***

[a] CFA : Confirmatory factor analysis

[b] Cronbach's alpha for the total group and with deletion of each of the individual items

*p<0.05, **p <0.01, ***p <0.001

Additionally, at T1 and T2, alpha were respectively 0.77 and 0.80. Factor loadings were all significant (p<0.01) and respectively between 0.67-0.82, and 0.67-0.83.

Graph S2.4. Multiple correspondence analysis graph representing the projection of the modalities of the items included in Attitudes latent variable - PRISME, France, 2019

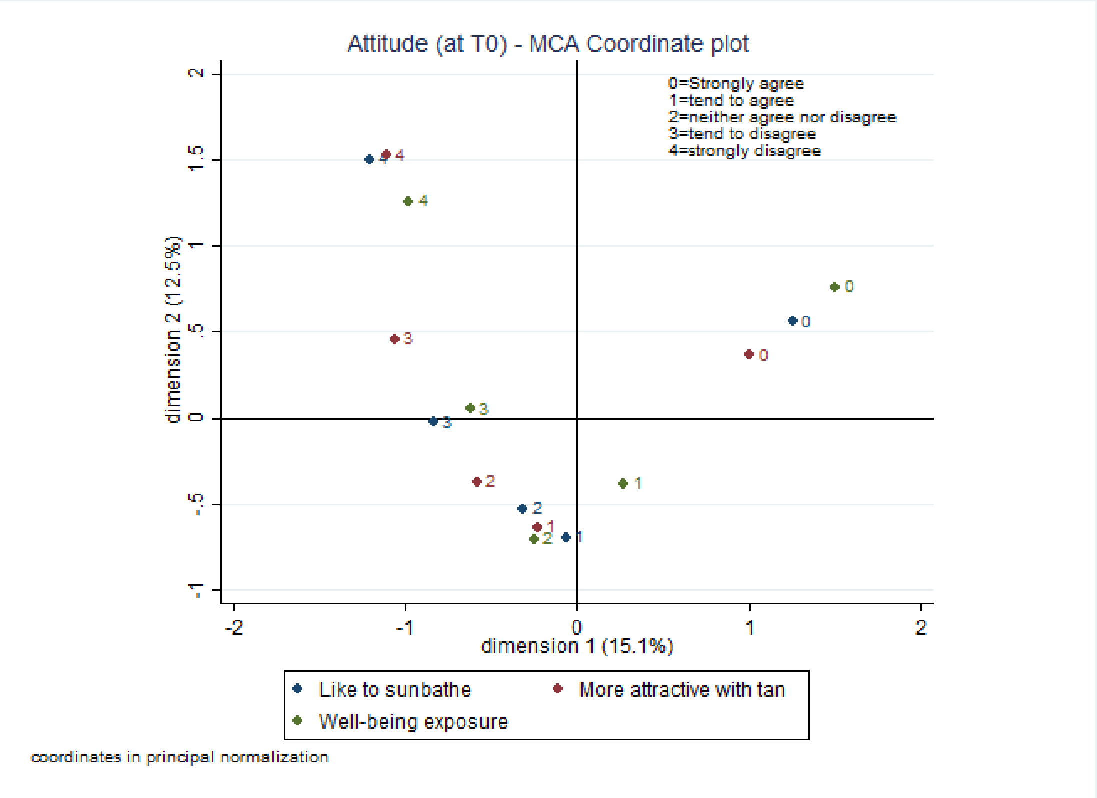

Social norm

Social norm was measured with two items on a reverse 5-point Likert scale (0=strongly disagree to 4=strongly agree): *"The people who I care about encourage me to protect myself from the sun"* (Encouragement of relatives), *"the people who I care about protect themselves from the sun"* (Protection of relatives).

Table S2.5. Internal consistency of the constructed latent variable 'Social Norm' in terms of Cronbach's alpha, correlation, and factor loading of the CFA[a] – PRISME, France, 2019

	N	Alpha[b]	Correlation with the rest	CFA[a] Factor loading
**Social Norm (at T0)**	**1355**	**0.54**		
Encouragement of relatives	1355		0.37	0.57*
Protection of relatives	1355			0.60*

[a] CFA : Confirmatory factor analysis

[b] Cronbach's alpha for the total group

*p<0.05, **p <0.01, ***p <0.001

Additionally, at T1 and T2, alpha were respectively 0.59 and 0.71. Factor loadings were all significant (p<0.05) and respectively 0.51-0.83, and 0.56-0.87.

Graph S2.5. Multiple correspondence analysis graph representing the projection of the modalities of the items included in Social Norm latent variable - PRISME, France, 2019

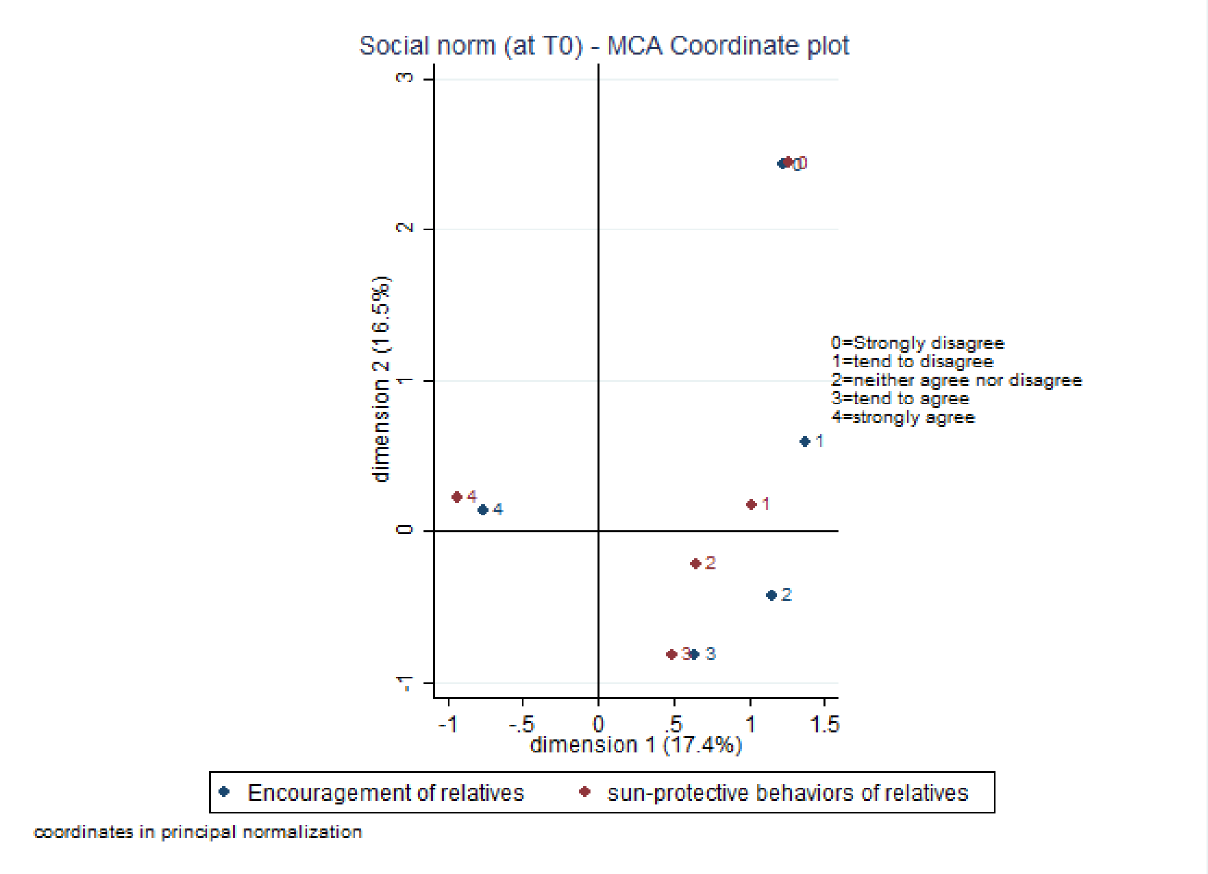

Goodness-of-fit for the CFA model was good: RMSEA=0.051, SRMR=0.064, with a coefficient of determination $R2=0.99$.

Conclusion

For each latent variable, although alpha was low to moderate, all the items were correlated and maximized the alpha coefficient (Tables S2.1-5).
MCA graphs (Graphs S2.1-5) confirmed that the responses of each item were close to one another.
In the CFA analysis, all factor loadings were significant and moderate in size, although some were below the 0.5 cut-off threshold (Tables S2.1-5).
The alphas and the factor loadings measured for each latent variable at the two other data collection times (T0 or T1, T2) were consistent, with close alphas and significant factor loadings.

References

1. Tavakol M, Dennick R. Making sense of Cronbach's alpha. International journal of medical education. 2011;2:53.
2. Truong Y, McColl R. Intrinsic motivations, self-esteem, and luxury goods consumption. Journal of Retailing and Consumer Services. 2011;18(6):555-61.
