# Supplementary material for: Influence of Social and Psychosocial Factors on Summer Vacationers’ Sun Protection Behaviors, the PRISME Study, France
Source: Int J Public Health. 2022 Aug 10;67:1604716. doi: 10.3389/ijph.2022.1604716 (PMC9399345; doi:10.3389/ijph.2022.1604716)
Supplement: Supplementary file 2 [file DataSheet1.pdf]

International Journal of Public Health

Influence of social and psychosocial factors on French summer vacationers' sun protection behaviors, the PRISME study, France

Supplementary file 1: Construction of skin sensitivity variable using a multiple correspondence analysis (MCA) followed by a hierarchical ascendant classification (HAC) – PRISME

1. Introduction

Skin sensitivity is an important physical characteristic to take into account when studying the sun protection of individuals. In order to evaluate it, six physical characteristics items were collected in our questionnaire: skin color, eye color, hair color, presence of moles, tendency to sunburn the day after critical exposure, and tendency to tan one week after critical exposure.

The most commonly used classification, especially during clinical evaluation in dermatology, is the Fitzpatrick phototype (1):
• Phototype I: very white / fair skin with often freckles, blond or red hair, blue or green eyes - Never tans and gets sunburns very easily.
• Phototype II: fair skin and sometimes has freckles, blond, red or brown hair, green / brown eyes - Barely tans and easily gets sunburns.
• Phototype III: medium light skin, chestnut or brown hair, brown eyes - Gradually tans and occasionally gets sunburns.
• Phototype IV: dark skin tone, brown / black hair, brown / black eyes - Tans easily and rarely gets sunburns.
• Phototype V: very dark skin, black hair, black eyes - Tans quickly and a lot, very rarely gets sunburns.
• Phototype VI: black skin, black hair, black eyes - Never gets sunburns.

From the answers to the six questionnaire items, it would have been difficult to categorize the participants according to this classification because few would perfectly correspond to all the physical characteristics of a phototype. To do that, it would be necessary to decide on a prioritization between these six characteristics, what we did not want to do because it led to a part of subjectivity in the method (2, 3).

In order to create the most homogeneous possible classes for these six characteristics using a statistical approach, we conducted a multiple correspondence analysis (MCA) followed by a hierarchical ascendant classification (HAC) using R-Studio version 1.3 (FactomineR).

The resulting skin sensitivity variable created was introduced as an explanatory and adjustment variable in the various models constructed in the PRISME study.

2. **Methods**

Multiple correspondence analysis (MCA)

The sample included 1355 individuals.

The MCA took into account six active variables containing 29 answer modalities (Table S1.1).

Table S1.1. Description of the active variables included for the construction of the skin sensitivity classification by MCA/HAC – PRISME, France, 2019

Items	Answers	N
q1 Declared skin color	1. Very light or white<br>2. Light or pale<br>3. Quite light to slightly golden<br>4. Light brown, matte, olive<br>5. Dark brown / Black	432<br>673<br>158<br>68<br>24
q2 Eye color	1. Light blue / light grey / light green<br>2. Blue / green / grey<br>3. Light brown or hazel<br>4. Dark brown<br>5. Brown-Black<br>Missing (*imputed*)	194<br>368<br>500<br>256<br>36<br>1
q3 Natural hair color	1. Red or light blond / blond<br>2. Dark blond or light brown<br>3. Chestnut<br>4. dark brown<br>5. Black	130<br>236<br>572<br>331<br>86
q4 Presence of moles	1. Many<br>2. Several<br>3. Few<br>4. Very few<br>5. None<br>Missing (*imputed*)	155<br>224<br>316<br>308<br>351<br>1
q5 Tendency to sunburn the day after critical exposure	1. Always<br>2. Often<br>3. Sometimes<br>4. Rarely<br>5. Never<br>Missing (*imputed*)	558<br>200<br>213<br>249<br>129<br>6
q6 Tendency to tan one week after critical exposure	1. No tan<br>2. Light tan<br>3. Medium tan<br>4. Dark tan<br>Missing (*imputed*)	71<br>596<br>479<br>194<br>15

Missing data were imputed using a 2-dimensional MCA model.

Hierarchical ascendant classification (HAC)

The number of dimensions of the MCA to be kept and the number of final classes to be constituted by the HAC was determined by varying the number of dimensions from 3 to 19 and by analyzing the histogram of inertia gain of the HAC.

Consolidation using the moving centers method was applied to the results of the HAC by iteratively reassigning each individual to the class for which his distance to the center of gravity of the class is

the shortest until optimization of the inertia gains. This consolidation increases the inter-class inertia and thus increases the relevance of the classification.

3. Results

Multiple correspondence analysis

Graph S1.1 (a) and (b). Graph of individuals (a) and projection of the modalities of the active variables (b) of the MCA - PRISME, France, 2019

(a) individuals

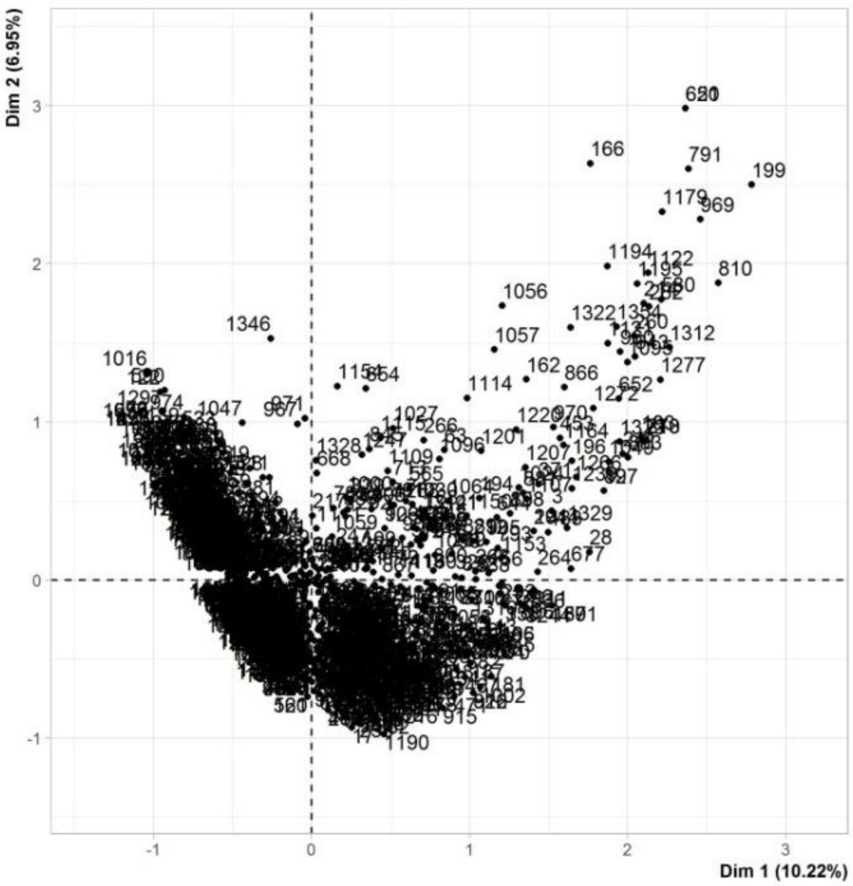

(b) Modalities

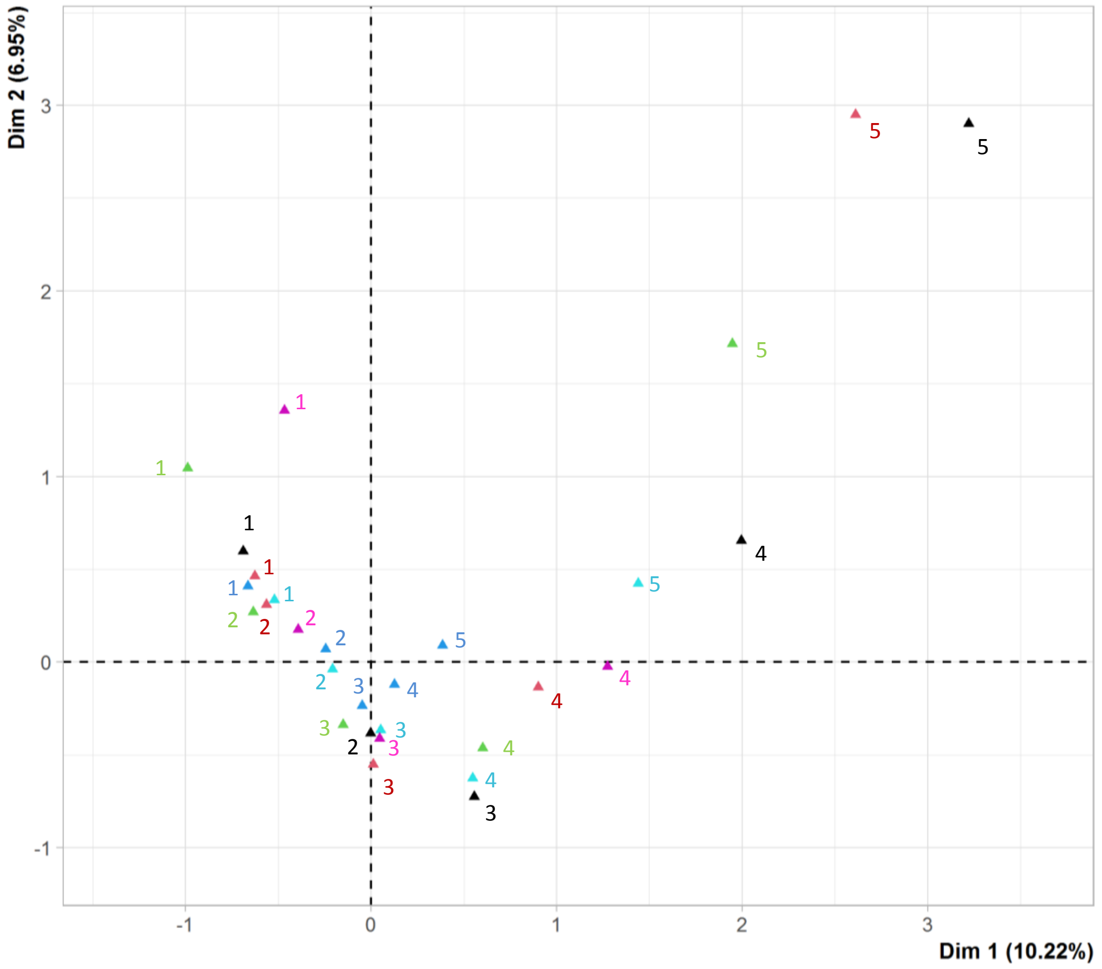

Legend:

▲ Declared skin color	1. Very light or white<br>2. Light or pale<br>3. Quite light to slightly golden<br>4. Light brown, matte, olive<br>5. Dark brown / Black	▲ Eye color	1. Light blue / light grey / light green<br>2. Blue / green / grey<br>3. Light brown or hazel<br>4. Dark brown<br>5. Brown-Black
▲ Natural hair color	1. Red or light blond / blond<br>2. Dark blond or light brown<br>3. Chestnut<br>4. dark brown<br>5. Black	▲ Presence of moles	1. Many<br>2. Several<br>3. Few<br>4. Very few<br>5. None
▲ Tendency to sunburn the day after critical exposure	1. Always<br>2. Often<br>3. Sometimes<br>4. Rarely<br>5. Never	▲ Tendency to tan one week after critical exposure	1. No tan<br>2. Light tan<br>3. Medium tan<br>4. Dark tan

The "extreme" individuals corresponding to darker to black skins are at the top right of the graph of individuals (Graph S1.1a). As is often the case in MCA, we observed the "Gutmann effect" on this graph in the form of a horseshoe. On axis 1 (Dimension 1), we observe an opposition between, on the

left side of the graph, people with red/blond hair, light eyes and a very light skin color, and on the right side of the graph, people with dark skin, black eyes and black hair (Graph S1.1b).

Graph S1.2. Active variables graph of the MCA - PRISME, France, 2019

t0_q1ter	Skin color
t0_q2bis	Eye color
t0_q3ter	Hair color
t0_q4bis	Moles
t0_q5bis	Tendency to burn
t0_q6bis	Tendency to tan

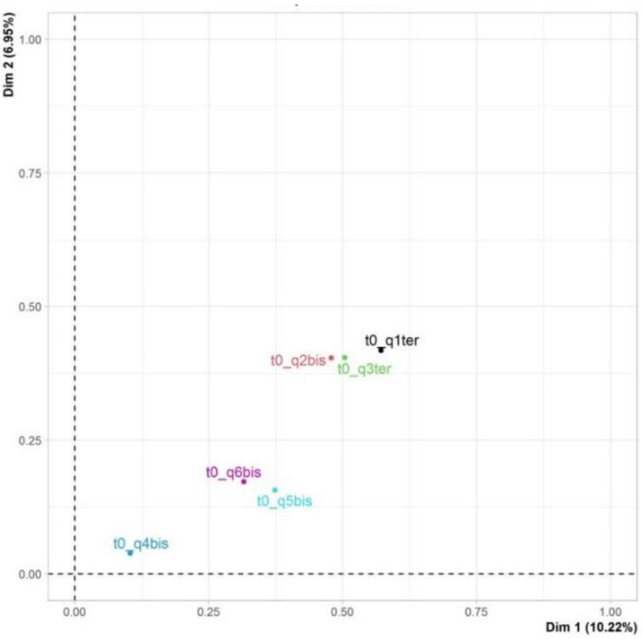

Table S1.2. Correlation (Eta coefficient) between active variables and the first three dimensions of the MCA- PRISME, France, 2019

	Dim. 1	Dim. 2	Dim. 3
Skin color	0.571	0.418	0.335
Eye color	0.478	0.404	0.267
Hair color	0.504	0.405	0.142
Moles	0.103	0.038	0.078
Tendency to burn	0.373	0.157	0.297
Tendency to tan	0.316	0.172	0.098

The presence of moles (q4) has less influence than the other variables in the construction of the first three axes (Graph S1.2, Table S1.2). The skin, eye and hair colors on the other hand have a strong influence on the first two axes.

Table S1.3. Coordinates (Dim), contribution (ctr), projection quality (cos2) and association (v. test) between the answers provided for the active variables and the first three dimensions of the MCA - PRISME, France, 2019

Variables	Answers	Dim. 1	ctr	cos2	v.test	Dim. 2	ctr	cos2	v.test	Dim. 3	ctr	cos2	v.test
	Very light	-0.69	6.46	0.22	-17.36	0.60	7.12	0.17	15.02	0.29	2.18	0.04	7.27
	Light	-0.01	0.00	0.00	-0.17	-0.38	4.56	0.14	-13.98	-0.44	7.94	0.19	-16.12
**Skin color**	Quite light	0.56	1.54	0.04	7.44	-0.72	3.84	0.07	-9.68	0.82	6.47	0.09	10.98
	Light brown, Matte	2.00	8.52	0.21	16.88	0.66	1.35	0.02	5.54	1.24	6.39	0.08	10.53
	Dark brown / Black	3.22	7.84	0.19	15.92	2.90	9.35	0.15	14.33	-1.76	4.53	0.06	-8.71
	Light blue/grey/green	-0.63	2.40	0.07	-9.44	0.46	1.94	0.04	6.98	-0.04	0.02	0.00	-0.55
	Blue/grey/green	-0.57	3.71	0.12	-12.72	0.31	1.64	0.04	6.97	0.25	1.37	0.02	5.56
**Eye color**	Light brown, Hazel	0.01	0.00	0.00	0.28	-0.55	7.03	0.18	-15.52	-0.34	3.51	0.07	-9.57
	Dark brown	0.90	6.55	0.19	16.01	-0.14	0.22	0.00	-2.42	0.65	6.49	0.10	11.48
	Brown-Black	2.61	7.74	0.19	15.89	2.95	14.52	0.24	17.95	-2.20	10.60	0.13	-13.39
	Red, light blond / blond	-0.99	4.01	0.10	-11.87	1.05	6.57	0.12	12.52	0.56	2.51	0.03	6.77
	Dark blond, light brown	-0.64	3.02	0.09	-10.77	0.27	0.79	0.02	4.55	0.01	0.00	0.00	0.24
**Hair color**	Chestnut	-0.15	0.42	0.02	-4.81	-0.34	3.02	0.08	-10.62	-0.31	3.33	0.07	-9.74
	Dark brown	0.60	3.77	0.12	12.59	-0.46	3.30	0.07	-9.71	0.45	4.13	0.07	9.48
	Black	1.95	10.25	0.26	18.65	1.71	11.70	0.20	16.42	-0.58	1.74	0.02	-5.53
	Many	-0.67	2.16	0.06	-8.81	0.41	1.21	0.02	5.43	0.42	1.69	0.02	5.61
	Several	-0.25	0.43	0.01	-4.03	0.07	0.05	0.00	1.17	0.09	0.11	0.00	1.50
**Moles**	Few	-0.05	0.02	0.00	-1.01	-0.23	0.80	0.02	-4.75	-0.12	0.25	0.00	-2.34
	Very few	0.13	0.15	0.01	2.50	-0.12	0.21	0.00	-2.43	-0.42	3.26	0.05	-8.33
	None	0.39	1.64	0.05	8.39	0.09	0.13	0.00	1.97	0.22	1.07	0.02	4.88
	Always	-0.52	4.82	0.19	-16.15	0.33	2.90	0.08	10.33	0.32	3.58	0.07	10.02
	Often	-0.21	0.27	0.01	-3.20	-0.04	0.01	0.00	-0.60	-0.85	8.71	0.13	-12.97
**Tendency to burn**	Sometimes	0.05	0.02	0.00	0.82	-0.37	1.32	0.03	-5.81	-0.68	6.01	0.09	-10.84
	Rarely	0.55	2.36	0.07	9.59	-0.63	4.53	0.09	-10.95	0.62	5.87	0.09	10.89
	Never	1.44	8.44	0.22	17.22	0.42	1.08	0.02	5.08	-0.17	0.23	0.00	-2.02
	No tan	-0.47	0.50	0.01	-4.09	1.36	6.14	0.11	11.83	0.26	0.31	0.00	2.31
**Tendency to tan**	light tan	-0.39	2.94	0.13	-12.96	0.18	0.87	0.03	5.81	-0.09	0.32	0.01	-3.07
	Medium tan	0.05	0.03	0.00	1.24	-0.41	3.80	0.10	-11.30	-0.21	1.33	0.03	-5.84
	Dark tan	1.27	9.99	0.28	19.26	-0.02	0.01	0.00	-0.36	0.72	6.08	0.09	10.82

Test values highlighted in red represent a significantly positive association and blue values a significantly negative association between the modalities of answers and the associated dimension.

The first axis represents the skin sensitivity gradient, while the second separates extreme sensitivity from average sensitivity (Table S1.3).

Graph S1.3. Histogram of variances and cumulative variance curve on each dimension of the MCA - PRISME, France, 2019

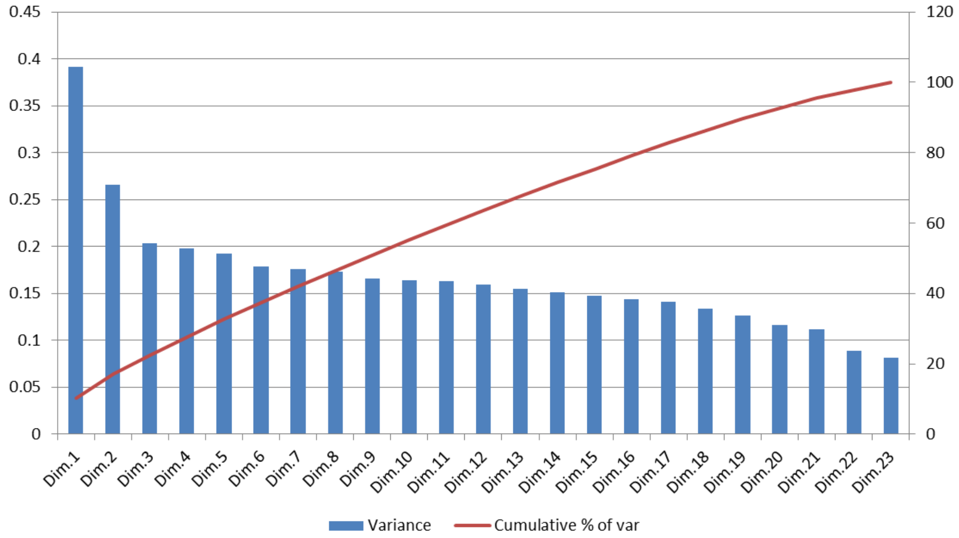

With a total variance of 3.83 (29 modalities of answers / 6 variables -1), the first axis represents 10.2% of the total variance and the second axis 6.9%. The variance decreases rapidly after axis 2 (Graph S1.3). The average variance is 0.166, so the first eight dimensions have a higher variance than the average.

Hierarchical ascendant classification

By varying the number of axes from 3 to 19, we see that over 10 dimensions the histogram of inertia gain is strongly modified with a jump in inertia between 2 and 3 classes that does not allow for an efficient class division (Graph S1.4). In order to minimize the loss of inter-class inertia, the choice of an 8-dimensional MCA allowing the creation of 4 classes during the HAC seems the most relevant.

Graph S1.4. Histogram of inertia gain of the HAC after MCA including 4/6/8/10/12 dimensions and number of classes that minimize the loss of inter-class inertia loss - PRISME, France, 2019

4 dimensions	6 dimensions	8 dimensions	10 dimensions	12 dimensions
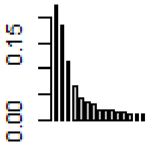 → 3 classes	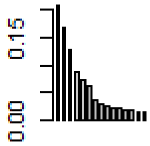 → 4 classes	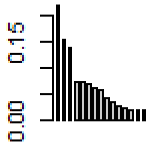 → 4 classes	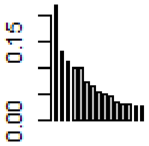 → 2 classes	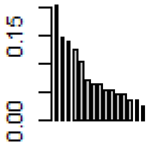 → 2 classes

The results of the HAC with these parameters were:

Graph S1.5. Cluster dendrogram of the HAC with four classes (after MCA including 8 dimensions) - PRISME, France, 2019

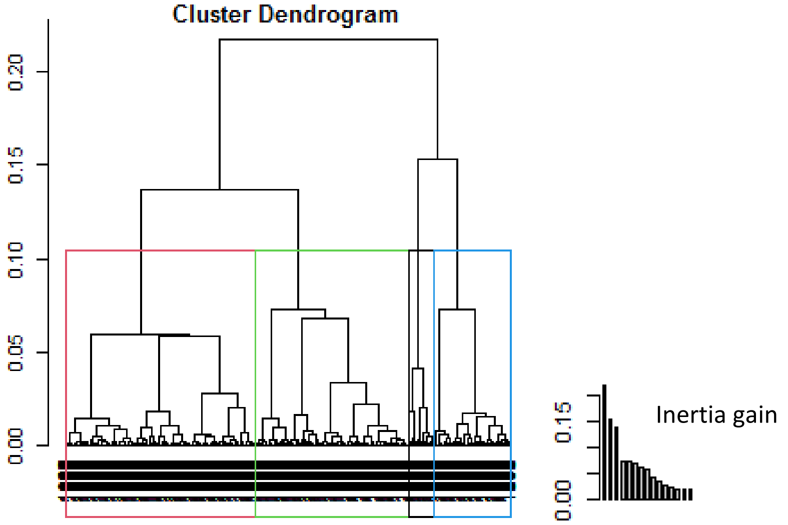

Graph S1.6. Factorial plan of the HAC with four classes (after MCA including 8 dimensions) on dimensions 1 and 2 - PRISME, France, 2019

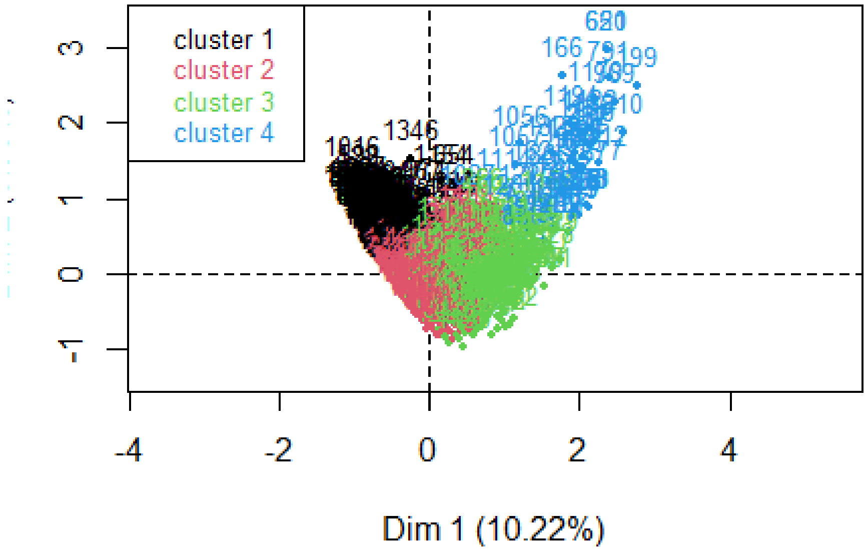

Table S1.4. Association between the answers of the active variables included in the MCA (8 dimensions) and the four classes of skin sensitivity created by the HAC - PRISME, France, 2019

		class 1	class 2	class 3	class 4
**Skin color**	Very light	23.1	-13.6	-10.1	-5.16
	Light	-14	22.2	-9.12	-4.79
	Quite light	-7	-7.8	16.6	-2.57
	Light brown, Matte	-7.29	-8.13	10.4	6.43
	Dark brown / Black	-4.07	-5.09	-2.51	12.7
**Hair color**	Red, light blond / blond	13	-8.74	-5.4	-2.89
	Dark blond, light brown	10.1	-4.61	-6.06	-3.57
	Chestnut	-4.38	7.03	-0.646	-6.99
	Dark brown	-11.4	4.15	6.74	1.51
	Black	-5.08	-4.65	2.7	11.5
**Eye Color**	Light blue/grey/green	8.85	-5.31	-3.14	-3.72
	Blue/grey/green	12.1	-6.57	-4.68	-5.58
	Light brown, Hazel	-9.95	10.7	0.696	-5.84
	Dark brown	-9.17	0.703	7.33	3.48
	Brown-Black	-4.51	-4.44	-3.22	13.8
**Moles**	Many	-3.33	-1.16	3.63	3.56
	Several	-5	6.8	-3.68	1.21
	Few	-0.859	0.727	1.25	-2.27
	Very few	3.44	-3.44	0.908	-1.5
	None	7.82	-4.3	-3.66	-2.53
**Tendency to burn**	Always	17.6	-9.6	-6.56	-6.41
	Often	-3.02	6.24	-4.54	-0.739
	Sometimes	-5.54	7.61	-3.26	-0.53
	Rarely	-9.24	1.86	7.75	0.0606
	Never	-8.6	-3.74	7.8	8.14
**Tendency to tan**	No tan	7.99	-6.35	-3.22	0.738
	light tan	9.54	-1.02	-9.24	-3.66
	Medium tan	-7.89	11.7	-4.84	-2.09
	Dark tan	-9.74	-11.2	18.5	6.06

Test values measure the discriminating power of each modality in the class. Values highlighted in red represent a significantly positive association (>=1.96) and blue values a significantly negative association (<=1.96) between the answer modalities and the class, which means that the proportion of individuals in this modality is higher or lower in this class than in the total population.

4. Conclusion

The associations found allowed us to describe the characteristics of the four skin sensitivity classes (Table S1.5).

Table S1.5. Description of the characteristics and numbers of the four classes created by the MCA /HAC - PRISME, France, 2019

Class of sensitivity		n	Skin color	Hair color	Eye color	Moles	Tendency to burn	Tendency to tan
**1**	**Highly sensitive**	456 (34%)	Very light	Red / Blond (light or dark) / Light brown	Blue/grey/green (light or not)	Many / Severals	Always	No/light tan
**2**	**Sensitive**	619 (46%)	Light	Chestnut / Dark brown	Light brown, Hazel	Very few	Often / sometimes	Medium tan
**3**	**Slightly sensitive**	226 (17%)	Quite light/ Matte	Dark brown/Black	Dark brown	None	Rarely / Never	Dark tan
**4**	**Dark to black skin**	54 (4%)	Matte / Dark brown / Black	Black	Dark brown / Brown-Black	None	Never	Dark tan

When comparing these characteristics with Fitzpatrick's phototype classification, class 1 appears to correspond to phototypes 1 and 2, class 2 to phototype 3, class 3 to phototype 4 and class 4 to phototypes 5-6.

In our sample of 1 355 participants, 34% belonged to class 1, 46% to class 2, 17% to class 3 and 4% to class 4. The proportions found are consistent with the distribution of phototypes described in the French Cancer Barometer of 2015 (phototype 1-2=32%; phototype 3=40%; phototype 4=24%; phototypes 5-6=4%) (4).
Moreover, participants of the PRISME study also had a measurement of their skin color on the inner side of the arm at inclusion (T0) with a colorimeter (5). If we describe this colorimetry measure (inside arm at t0) according to the four classes of skin sensitivity, we obtain values consistent with this classification (Table S1.6).

Table S1.6. Distribution of ITA* on the inner side of the arm at baseline in the four classes of skin sensitivity created by the MCA/HAC - PRISME, France, 2019

Class	Mean (SD)	Median	P25-P75	Min-max
1	49.4 (12.8)	51.5	43.0 – 58.5	2.5 – 76.5
2	39.9 (12.9)	42.5	31.0 – 48.5	-17.5 – 66.5
3	30.0 (14.0)	30.5	22.0 – 40.9	-13.0 – 64.0
4	0.2 (31.3)	0.0	-15.8 – 26.0	-77.0 – 61.5

**ITA = Individual topological angle is a measure of skin color by colorimetry. The lower the ITA, the darker the skin.*

References

1. Fitzpatrick TB. The validity and practicality of sun-reactive skin types I through VI. Arch Dermatol. 1988;124(6):869-71.
2. Holm-Schou AS, Philipsen PA, Wulf HC. Skin cancer phototype: A new classification directly related to skin cancer and based on responses from 2869 individuals. Photodermatol Photoimmunol Photomed. 2019;35(2):116-23.
3. Trakatelli M, Bylaite-Bucinskiene M, Correia O, Cozzio A, De Vries E, Medenica L, et al. Clinical assessment of skin phototypes: watch your words! Eur J Dermatol. 2017;27(6):615-9.
4. Ménard C, Thuret A. Baromètre cancer 2015. Ultraviolets, naturels ou artificiels. Connaissance, croyances et pratiques de la population en 2015. Saint-Maurice : Santé publique France. 2018:46.
5. Durand C, Catelinois O, Bord A, Richard J-B, Bidondo M-L, Ménard C, et al. Effect of an Appearance-Based vs. a Health-Based Sun-Protective Intervention on French Summer Tourists' Behaviors in a Cluster Randomized Crossover Trial: The PRISME Protocol. Front Public Health. 2020;8(654).
